# Proteome profiles during early stage of somatic embryogenesis of two *Eucalyptus* species

**DOI:** 10.1186/s12870-022-03956-4

**Published:** 2022-12-02

**Authors:** Bowen Chen, Changrong Li, Yingying Chen, Shengkan Chen, Yufei Xiao, Qi Wu, Lianxiang Zhong, Kaiyong Huang

**Affiliations:** Guangxi Key Laboratory of Superior Timber Trees Resource Cultivation, Guangxi Forestry Research Institute, 23 Yongwu Road, Nanning, 530002 Guangxi China

**Keywords:** Somatic embryogenesis, Dedifferentiation, *Eucalyptus*, Proteomics, Regeneration, Propagation

## Abstract

**Background:**

Somatic embryogenesis (SE) was recognized as an important tool for plants to propagate. However, our knowledge about the proteins involved in early SE including the callus dedifferentiation is still limited, especially in the economic woody tree – *Eucalyptus*.

**Results:**

We used the data-independent acquisition mass-spectrometry to study the different proteome profiles of early SE of two *Eucalyptus* species—*E. camaldulensis* (high regeneratively potential) and *E. grandis* x *urophylla* (low regenerative potential). Initially, 35,207 peptides and 7,077 proteins were identified in the stem and tissue-culture induced callus of the two *Eucalyptus* species. MSstat identified 2,078 and 2,807 differentially expressed proteins (DEPs) in early SE of *E. camaldulensis* and *E. grandis* x *urophylla*, respectively. They shared 760 upregulated and 420 downregulated proteins, including 4 transcription factors, 31 ribosomal proteins, 1 histone, 3 zinc finger proteins (ZFPs), 16 glutathione transferases, 10 glucosyltransferases, ARF19, WOX8 and PIN1. These proteins might be involved in the early SE of *Eucalyptus*. By combining the miRNA and RNA-Seq results, some miRNA ~ gene/protein regulatory networks were identified in early SE of *Eucalyptus*, such as miR160 ~ TPP2, miR164 ~ UXS2, miR169 ~ COX11 and miR535 ~ Eucgr.E01067. Further, we found SERK, WRKY, ZFP and ABC transporter might be related with high SE potential.

**Conclusions:**

Overall, our study identified proteins involved in the early SE and related to the high regeneration potential of *Eucalyptus*. It greatly enhanced our understanding of the early SE and the SE capacity of *Eucalyptus*.

**Supplementary Information:**

The online version contains supplementary material available at 10.1186/s12870-022-03956-4.

## Background

Somatic embryogenesis (SE) is an intricate molecular and biochemical process that asexual embryos emerge from somatic cells or vegetative tissues without fertilization. The somatic embryos can be formed directly or indirectly from the callus tissues which are induced from vegetative tissues of plants. Since Steward firstly reported the asexual embryogenesis in the cell suspension cultures of carrot in 1958, in vitro SE systems have been established in many plant species [1]. For *Eucalyptus* – a highly diverse genus of the Myrtaceae family widely planted across the world for its increasing importance for timber and pulp [2, 3], Ouyang et al. reported the SE from the callus of seedlings for the first time in 1980 [4]. Early SE is the callus induction in which differentiated somatic cells (e.g., seed, leaf, stem) acquire embryogenetic competence with or without a dedifferentiation step [5]. Several characteristic events have been reported to happen in early SE, including dedifferentiation of cells, activation of cell division and reprogramming of cell physiology, metabolism and gene expression patterns [6].

Some protein and genes have been reported to function as regulatory factors at different SE stages. For example, ARF19 (auxin response factor 19), PRC1 (polycomb repressive complex 1), RGP1 (UDP-arabinopyranose mutase 1) and HSP17 (heat shock protein 17) are involved in the dedifferentiation; SERK1 (somatic embryogenesis like receptor kinase 1), LEC1 (leafy cotyledon 1, also called as nuclear transcription factor Y subunit B-9), GLB1 (galactosidase beta 1), WUS (WUSCHEL) and CLF (curly leaf) are involved in the acquisition of totipotency by the cells; and CDKA1 (cyclin-dependent kinase A-1), PRZ1 (transcriptional regulator prz1) and STM (homeobox protein SHOOT MERISTEMLESS) are involved in the commitment of totipotent cells to embryogenic state [7]. In addition, some inductive signals conducive to de-differentiation have been identified, including the plant growth regulators, heavy metals, and the imposition of stress conditions (e.g., high temperature, osmotic shock, or water stress) [8]. In *Arabidopsis*, Elhiti and colleagues summarized the functions of 51 proteins involved in early SE [9]. Recently, plant hormone signalling-related genes, especially the auxin and cytokinin signalling components, were reported to be significantly enriched in early SE of hybrid sweetgum (*Liquidambar styraciflua* × *Liquidambar formosana*) and four modules of genes were identified with properties relating to embryonic potential, early somatic embryogenesis, and somatic embryo development [10]. Core cell cycle genes, cytochrome P450 genes and polyamines have been shown to play an important regulatory role during the early SE of *Dimocarpus longan* Lour [11–13]. However, our knowledge about *Eucalyptus* proteins involved in early SE is limited.

Proteomics technologies enable the researchers to identify hundreds of proteins associated to the SE process in plants. Since 2000, more than 100 studies have used the 2DE-based mass spectrometry (MS) proteomics approaches to investigate the proteome profiles of SE in plants [14]. For example, Pan et al*.* identified 24 differentially expressed proteins (DEPs) involved in the SE of *Citrus sinensis* Osbeck [15]; and Sun et al. identified 29 DEPs between the embryogenic calli and non-embryogenic calli by using the 2DE-based MS and they were involved in the cell proliferation (10.34%), transcription and protein processing (17.24%), stress response (10.34%), signal transduction (3.45%), metabolism and energy (48.28%) and hypothetical function (10.34%) [16]. While the plant proteomics is processing from 2DE-based approaches to shotgun proteomics which applies high-throughput gel-free approaches, including DDA (data-dependent acquisition) and DIA (data-independent acquisition), and allows the identification of low abundant proteins in the samples [14]. Many researchers have employed the gel-free proteomics platforms to identify SE related proteins in different plant species, such as *Araucaria angustifolia* [17], *Coffea arabica* [18], *Gossypium hirsutum* [19], *Picea asperata* [20], *Picea balfouriana* [21], *Saccharum* spp. [22] and *Zea mays* [23]. Notably, proteomics technologies also enable the identification of gene/protein regulation networks in the SE process of plants [24]. Interestingly, not many shotgun proteomics studies were demonstrated for *Eucalyptus* [25–27].

Previously, our lab published the transcriptome and miRNA profiles of tissue-culture induced callus of two *Eucalyptus* species—*E. camaldulensis* (high regeneratively potential) and *E. grandis* x *urophylla* (low regenerative ability) [2, 28, 29]. We found that early stage of SE is important for downstream callus development as it prepares for the acquisition of SE potential of callus via dedifferentiation on transcriptional level [2]. We have uncovered some genes involved in this process, such as genes whose products are related to SERK, ethylene, auxin, ribosomal protein (RP), zinc finger protein (ZFP), heat shock protein (HSP), histone, cell wall and transcription factor (TF) [2, 29]. Further, some miRNA-gene pairs like MIR160 ~ ARF18, MIR396 ~ GRF6, MIR166 ~ ATHB15/HD-ZIP and MIR156/MIR157 ~ SPL1 were identified in the SE of these two *Eucalyptus* species [28]. In the present study, we aimed to investigate the proteome profiles of stem and callus tissues during the early SE of the two *Eucalyptus* species using the DIA MS approach. The DEPs will be analysed to uncover proteins involved in the early SE and related to the high SE potential. This is the first time to investigate the proteome profiles of early SE in *Eucalyptus* and the findings will improve our understanding of the molecular changes and regulatory networks in this process.

## Materials and methods

### Plant samples and treatment

The original seeds of high regenerative ability *Eucalyptus* species (*E. camaldulensis*, voucher ID: c0009) and low regenerative ability *Eucalyptus* species (*E. grandis* x *urophylla*, voucher ID: j0017) were obtained from the wild in 1984, and no permissions were required to collect these plants. The seeds and plants were confirmed by a senior botanist Prof. Dongyun Xiang. The seedlings of the *Eucalyptus* species were kept in the Eucalyptus Resource Garden of Guangxi Forestry Research Institute (Naning, China). The second generation of in vitro tissue-culture induced seedlings of both *Eucalyptus* species were maintained on the MS medium (supplemented with 20 mg/L Ca(NO_3_)_2_, 0.5 mg/L 6-BA and 0.1 mg/L IAA) until 2 to 3 cm height. The second to the third stems from the stem tip of the seedlings were obtained and cut into 0.3 ~ 0.5 cm segments. For each *Eucalyptus* species, about 60 segments were collected and transferred onto the induction MS medium (supplemented with 20 mg/L Ca(NO_3_)_2_, 1 mg/L KT and 0.5 mg/L 2,4-D) and maintained in darkness at 28 ± 2 °C for 10 days. The callus tissues were weighted every day to perform the growth curve analysis, as described [2]. Stem (0 d) and primary callus (10 d) tissues were used as differentiated (stem, A1 for *E. camaldulensis*, B1 for *E. grandis* x *urophylla*) and dedifferentiated (callus, A2 for *E. camaldulensis*, B2 for *E. grandis* x *urophylla*) samples, respectively. The induction experiment was replicated three times.

### Protein extraction

We used 100 mg of stem and callus tissues for the protein extraction. First, we added ~ 1 mL 1 × Cocktail (with EDTA but without SDS L3) to the plant samples and placed them on ice for 5 min. After DTT was added and diluted to the final concentration of 10 mM DTT, the plant cells were lysed by a sonicator, centrifuged at 25,000 g for 15 min at 4 °C. Then, the supernatant was collected and DTT was added to the supernatant to 10 mM of DTT. After the sample was water bathed at 56 °C for 1 h, IAM was added to the sample until the concentration of 10 mM IAM, followed by an incubation in darkness for 45 min. After a centrifugation at 25,000 g for 15 min at 4 °C, the supernatant was taken as the protein solution.

### Protein quant and quality control

Bradford quantification and SDS-PAGE were used for the protein quant and quality control, according to the manufacturers’ instructions.

### Protein digestion and high pH RP separation

Protein solution (100 μg) per sample was diluted with 50 mM NH_4_HCO_3_ by 4 times volume and added with Trypsin enzyme (2.5 μg, 1/40 volume of the protein solution) for digestion for 4 h at 37 °C. Then, the tryptic peptides were acidified with 1% formic acid (FA) and centrifuged at 12,000 g for 5 min at room temperature. The supernatant was desalted using a Strata X column and vacuumed to dryness. Then, the peptides were separated using a Shimadzu LC-20AB HPLC system coupled with a Gemini high pH C18 column (5 μm, 4.6 × 250 mm). Equal amount of the peptides from all samples were mixed, diluted with mobile phase A (5% ACN, 95% H_2_O, pH 9.8 adjusted with ammonia), injected to the column and eluted at a flow rate of 1 mL/min by gradient: 5% mobile phase B (95% ACN, 5% H_2_O, pH 9.8 adjusted with ammonia) for 10 min, 5% to 35% mobile phase B for 40 min, and 35% to 95% mobile phase B for 1 min. Then, the system was maintained in 95% phase B for 3 min, followed by a decrease to 5% phase B within 1 min and equilibration with 5% phase B for 10 min. The elution peak was monitored at an absorbance of 214 nm and fractions were collected every minute. The eluted peptides were pooled as 10 fractions and freeze-dried.

### DDA spectrum library construction

The dried peptides were reconstituted with mobile phase A (2% ACN, 0.1% FA) and centrifuged at 20,000 g for 10 min. The supernatant was taken for injection in a Thermo UltiMate 3000 UHPLC liquid chromatograph. In brief, the sample was enriched in the trap column, desalted, and entered a tandem self-packed C19 column (150 μm internal diameter, 1.8 μm column size, 35 cm column length). The peptides were separated at a flow rate of 500 nL/min by the following effective gradient: 0 ~ 5 min with 5% phase B (98% ACN, 0.1% FA), 5 ~ 120 min with phase B increased from 5 to 25%, 120 ~ 160 min with phase B from 25 to 35%, 160 ~ 170 min with phase B from 35 to 80%, 170 ~ 175 min with 80% phase B, 175 ~ 180 min with 5% phase B. The nanoliter liquid phase separation end was directly connected to the mass spectrometer as the following settings. The LC separated peptides were then ionized by nanoESI and injected to tandem mass spectrometer Q-Exactive HF X (Thermo Fisher Scientific) with data-dependent acquisition (DDA) mode. The major settings were applied as follows: ion source voltage 1.9 kV; MS scan range 350 ~ 1,500 m/z; MS resolution 120,000, maximal injection time (MIT) 100 ms; MS/MS collision type HCD, collision energy NCE 28; MS/MS resolution 30,000, MIT 100 ms, dynamic exclusion duration 30 s. The initial m/z for MS/MS was set to 100. Precursor for MS/MS scan satisfied: charge range 2 + to 6 + , top 20 precursors with intensity over 5e4. AGC for MS and MS/MS scan was set to 3e6 and 1e5, respectively.

### LC–MS analysis in DIA mode

For data-independent acquisition (DIA) analysis, the LC separated peptides of each sample were ionized by nanoESI and injected to the tandem mass spectrometer Q-Exactive HF X (Thermo Fisher Scientific) with DIA mode. The following settings were applied for the DIA analysis: ion source voltage 1.9 kV; MS scan range 400 ~ 1,250 m/z, MS resolution 120,000, MIT 50 ms; 400 ~ 1250 m/z was equality divided to 50 continuous windows for MS/MS scan. The MS/MS collision type was HCD, and MIT was set to auto mode. The fragment ions were scanned in Orbitrap with MS/MS resolution 30,000, collision energy in distributed mode (22.5, 25, 27.5) and AGC 1e6.

### Database searching and protein quantitation

DDA MS raw files were analysed using the Andromeda search engine within MaxQuant (v1.5.3.30) and the NCBI non-redundant *Eucalyptus* protein sequences (44,589 sequences) were used as the reference [30]. The search parameters were set as follows: cutting enzyme trypsin; minimal peptide length 7; PSM-level false discovery rate (FDR) 0.01; protein FDR 0.01; fixed modification carbamidomethyl (C); variable modifications oxidation (M) and acetyl (protein N-term). The identified results were used for the spectral library construction for DIA analysis. The DIA data was analysed using the iRT peptides for retention time calibration. Then, mProphet algorithm was used to complete the analytical quality control. Significant and reliable quantitative results were obtained based on the target-decoy model applicable to SWATH-MS with FDR 0.01.

### Bioinformatics analysis

We annotated the *Eucalyptus* protein sequences by mapping them against the Gene Ontology (GO), Cluster of Orthologous Groups (COG) and Kyoto Encyclopedia of Genes and Genomes (KEGG) pathway databases [31, 32]. Then, DEPs in differentiated and dedifferentiated *Eucalyptus* tissues were identified by MSstats with following criteria: log2 fold change (log2FC) > 1 or < -1, adjusted *p*-value < 0.05 [33]. Next, enriched GO terms and KEGG pathways by the DEPs were identified by *p*-value (< 0.05) calculated by Fisher’s exact test, and *q*-value (< 0.05), calculated by the R package ‘*q* value’.

### qRT-PCR validation

We selected 5 miRNAs (egd-N-miR278-5p. egd-miR395a-3p. egd-N-miR230-5p, egd-miR169r-5p and egd-miR160c-5p) and 10 genes (Eucgr.I02222, Eucgr.L01850, Eucgr.F01428, Eucgr.F03955, Eucgr.I02364, Eucgr.B01758, Eucgr.H03408, Eucgr.E00258, Eucgr.I01125 and Eucgr.A02778) whose protein products were differentially expressed during the early SE of *Eucalyptus* for qRT-PCR validation. The actin gene (Eucgr.G02932, XP_010067406.1) was used as internal control. For the miRNA qRT-PCR experiment, we used the stem-loop methods and predicted the forward and reverse primers with miRPrimer2 [34]. While for the genes, we predicted the primers using Primer3 [35]. After the Ct values were calculated and averaged, we used the ΔCt value to present the gene expression in each sample (relative to actin). Then, the stem tissues (A1 and B1) were used as control to calculate the expression changes (ΔΔCt) of miRNAs/genes in the callus tissues (A2 and B2). Relative normalized expression (RNE) was used to show the gene expression change: RNE = 2^−ΔΔCt^ and log2RNE was used to match the DIA/small RNA sequencing results. Triplicate reactions were performed for a miRNA/gene in one sample. We used Student’s t-Test in R software to calculate the significance (*p*-value) of a candidate miRNA/protein in the comparison. The plots were generated by ggplot2 package in R software and error bars were present for the mean ± SD (standard deviation) of log2RNE values.

## Results

### DIA proteomics during early somatic embryogenesis of *Eucalyptus*

**Fig. 1:**
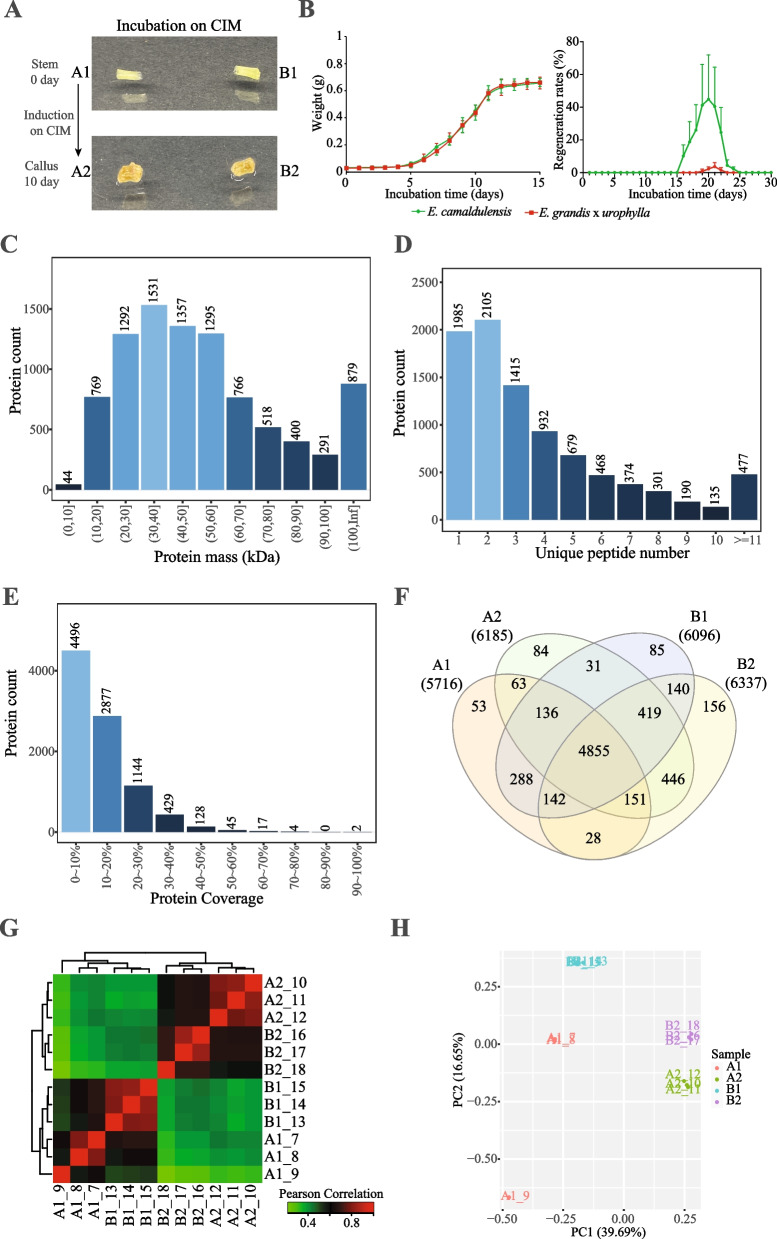
Overview of DIA proteomics for early somatic embryogenesis of two *Eucalyptus* species. (**A**) morphological characterization of stem (upper panel) and tissue-culture induced callus (lower panel) tissues of two *Eucalyptus* species. Left: *E. camaldulensis*; right: *E. grandis* x *urophylla*. (**B**) Growth curves of the tissue-culture induced callus of two *Eucalyptus* species on CIM. (**C**) Molecular weight of proteins identified in the early SE of *Eucalyptus*. (**D**) Distribution of unique peptides aligned to the *Eucalyptus* proteins. (**E**) Protein coverage by the DIA proteomics. (**F**) Venn diagram of proteins identified in the stem and callus tissues of *E. camaldulensis* and *E. grandis* x *urophylla*. (**G**) Heat map of sample correlation based on the protein expression data. (**H**) Principal component analysis of the stem and callus samples of *Eucalyptus*. A1: stem tissue of *E. camaldulensis*; A2: callus tissue of *E. camaldulensis*; B1: stem tissue of *E. grandis* x *urophylla*; B2: callus tissue of *E. grandis* x *urophylla*

Figure 1A showed the morphology characterization of stem tissues and tissue-culture induced callus of the two *Eucalyptus* species. Then, we found that 7 to 12 days were the rapid growth period for the stem tissue under CIM induction and we chose samples after 10 days induction as the primary callus to study the early SE of *Eucalyptus* (Fig. 1B, left panel). In addition, the regeneration rate of *E. camaldulensis* callus was found to be much higher than that of *E. grandis* x *urophylla* (Fig. 1B, right panel). The stem tissues and tissue-culture induced calli were then processed for the DIA proteomics. Initially, the DDA analysis produced 44,451 peptides corresponding to 9,142 proteins. Figure 1C showed that the molecular weight of 1,531, 1,357, 1,295 and 1,292 proteins was in 30 ~ 40 kDa, 40 ~ 50 kDa, 50 ~ 60 kDa and 20 ~ 30 kDa, respectively. Then, we found 447 and 2,105 proteins mapped by more than 10 and 2 unique peptides, respectively (Fig. 1D). We also found that 4,496 (49.17%) *Eucalyptus* proteins were covered less than 10% by the identified peptides and that two proteins covered more than 90% (Fig. 1E).

Next, we analysed the protein profiles in each sample using the LC–MS-DIA mode. Initially, 35,207 peptides corresponding to 7,077 proteins (A1: 5,716; A2: 6,185; B1: 6,096; B2: 6,337) were identified. Venn diagram (Fig. 1F) showed that the four samples shared 4,855 proteins and that 53, 84, 85 and 156 proteins were specifically identified in A1, A2, B1 and B2, respectively. We next evaluated the sample correlation using the protein expression profiles. A heat map of Pearson correlation revealed a linear correlation between 0.22 to 0.83 between the replicates of each sample (Fig. 1G). Notably, it showed that *Eucalyptus* dedifferentiated callus (A2 and B2) had distinct protein profiles from the differentiated stem tissues (A1 and B1). The principal component analysis confirmed the repeatability of biological replicates and the separation between stem and callus tissues (Fig. 1H).

### DEPs in the early somatic embryogenesis of *E. camaldulensis*

**Fig. 2:**
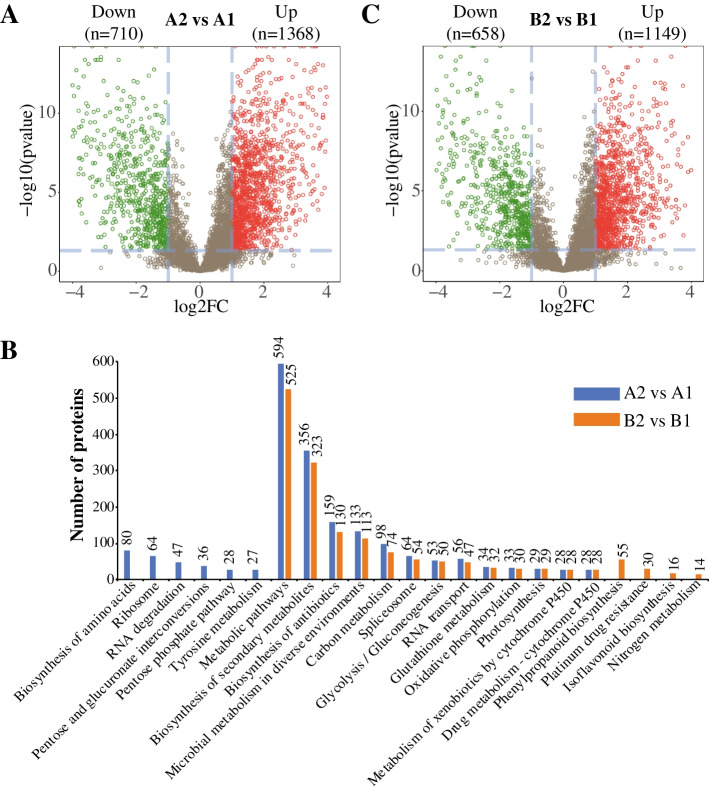
Differentially expressed proteins in the early SE of *Eucalyptus*. (**A**) Volcano plot of DEPs in the early SE of *E. camaldulensis*. (**B**) KEGG pathway enrichment of DEPs in the early SE of two *Eucalyptus* species. (**C**) Volcano plot of DEPs in the early SE of *E. grandis* x *urophylla*. A1: stem tissue of *E. camaldulensis*; A2: callus tissue of *E. camaldulensis*; B1: stem tissue of *E. grandis* x *urophylla*; B2: callus tissue of *E. grandis* x *urophylla*

We next identified 2,078 (1,368 upregulated and 710 downregulated proteins) DEPs during the early SE of *E. camaldulensis* (Fig. 2A, Table S1). Among them, we identified some protein families with interest, such as TF (11 upregulated and 5 downregulated), RP (8 upregulated and 43 downregulated), histone (1 upregulated and 4 downregulated), zinc finger protein (10 upregulated), ABC transporter protein (9 upregulated and 1 downregulated), glutathione transferase (19 upregulated and 3 downregulated), glucosyltransferase (18 upregulated and 2 downregulated) (Table 1). Notably, 1 BIM1, 1 bZIP, 1 WRKY, 1 GATA and 2 TGA2.3 TFs were upregulated in the early SE of *E. camaldulensis* (Table S1). XP_010060808.1 (glutathione S-transferase) was the most upregulated protein in the early SE of *E. camaldulensis* (Table S1). The deregulation of these proteins in early SE indicated their potential function in this process. Next, we analysed the GO and KEGG pathway enrichment by the DEPs in the early SE of *E. camaldulensis*. Table S2 showed that the DEPs were enriched in the biological processes of “cell wall modification” (GO:0,042,545, 7 proteins, *q*-value = 6.10E-04), “RNA modification” (GO:0,009,451, 6 proteins, *q*-value = 1.66E-02) and “photosystem II repair” (GO:0,010,206, 2 proteins, *q*-value = 1.76E-05). We also found 349, 117 and 102 DEPs from the “integral component of membrane” (GO:0,016,021) and “cytoplasm” (GO:0,005,737) and “nucleus” (GO:0,005,634). The KEGG pathway enrichment (Fig. 2B) analysis revealed that 594, 356 and 159 DEPs were involved in the pathways of “metabolic pathways”, “biosynthesis of secondary metabolites”, and “biosynthesis of antibiotics”, respectively.

**Table 1:** Potential protein families involved in the early SE and associated with the SE potential of *Eucalyptus*

Type	*E. camaldulensis*	*E. grandis* x *urophylla*	Shared	Only in *E. camaldulensis*	Only in *E. grandis* x *urophylla*
transcription factor	11/5	9/4	2/2	9/3	7/2
ribosomal protein	8/48	7/29	6/25	2/18	¼
histone	1/4	0/1	0/1	1/3	0/0
zinc finger protein	10/0	6/0	3/0	7/0	3/0
ABC transporter protein	9/1	8/1	3/0	6/1	5/1
glutathione transferase	19/3	18/3	15/1	4/2	3/2
glucosyltransferase	18/2	17/1	9/1	9/1	8/0

### DEPs in the early somatic embryogenesis of *E. grandis* x *urophylla*

We next identified 1,149 upregulated and 658 downregulated proteins in the dedifferentiated callus of *E. grandis* x *urophylla*, compared to the stem tissue (Fig. 2C, Table S3). Among them, 9 TF (5 upregulated and 4 downregulated), 36 RP (7 upregulated and 29 downregulated), 1 histone (1 downregulated), 6 zinc finger protein (6 upregulated), ABC transporter protein (8 upregulated and 1 downregulated), 21 glutathione transferase (18 upregulated and 3 downregulated), and 18 glucosyltransferase (17 upregulated and 1 downregulated) were identified (Table 1). The GO enrichment analysis showed that only 1 DEP was significantly involved in the biological process of “lateral root development” (GO:0,048,527) and that 304 and 26 DEPs were enriched in the “integral component of membrane” (GO:0,016,021) and “chloroplast thylakoid membrane” (GO:0,009,535), respectively (Table S4). Further, KEGG pathway analysis revealed most pathways enriched by the DEPs were shared by the two *Eucalyptus* species in the SE process. Like *E. camaldulensis*, the top three pathways enriched by the DEPs were “metabolic pathways” (525 DEPs), “biosynthesis of secondary metabolites” (323 DEPs), and “biosynthesis of antibiotics” (130 DEPs). Interestingly, we found some pathways specific to each *Eucalyptus* species (Fig. 2B). For example, 6 pathways, including “pentose and glucuronate interconversions” and “pentose phosphate pathway”, were specifically enriched by the DEPs in the SE of *E. camaldulensis*; 4 pathways including “phenylpropanoid biosynthesis”, “platinum drug resistance”, “isoflavonoid biosynthesis” and “nitrogen metabolism” were specifically enriched by the DEPs in the SE of *E. grandis* x *urophylla*.

### Early somatic embryogenesis associated proteins

**Fig. 3:**
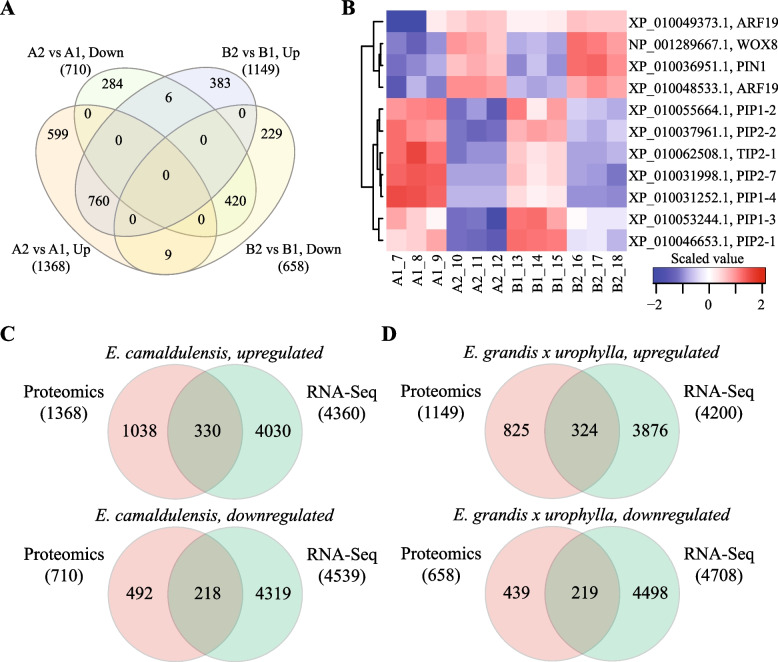
Somatic embryogenesis potential associated proteins for *Eucalyptus*. (**A**) Venn diagram of DEPs identified in early SE of the two *Eucalyptus* species. (**B**) Heat map of commonly deregulated proteins in the early SE of the two *Eucalyptus* species, including ARF19, WOX8, PIN1 and aquaporins. (**C**) Venn diagram of DEPs and differentially expressed genes by transcriptome sequencing in *E. camaldulensis*. (**D**) Venn diagram of DEPs and differentially expressed genes by transcriptome sequencing in *E. grandis* x *urophylla*. A1: stem tissue of *E. camaldulensis*; A2: callus tissue of *E. camaldulensis*; B1: stem tissue of *E. grandis* x *urophylla*; B2: callus tissue of *E. grandis* x *urophylla*

We next compared the DEPs in the two *Eucalyptus* species. Venn diagram (Fig. 3A) showed that 760 upregulated and 420 downregulated proteins were shared by *E. camaldulensis* and *E. grandis* x *urophylla* in the early SE process. Notably, 4 TFs (2 upregulated and 2 downregulated), 31 RPs (6 upregulated and 25 downregulated), 1 histone (downregulated), 3 ZFPs (3 upregulated), 3 ABC transporter (3 upregulated), 16 glutathione transferase (15 upregulated and 1 downregulated), and 10 glucosyltransferase (9 upregulated and 1 downregulated) were commonly deregulated in the two *Eucalyptus* species during SE (Table 1). The 4 TFs included At3g04930, ASR3, RAP2-1 and NFYB3. It is notable that WOX8 (WUSCHEL-related homeobox 8, NP_001289667.1) was upregulated in the early SE of the two *Eucalyptus* species. In addition, we found ARF19, PIN1 and aquaporins commonly deregulated in the two *Eucalyptus* species (Fig. 3B). Among them, all the aquaporin proteins were downregulated, and two ARF19 proteins and three ABC transporter proteins were upregulated in the early SE of the two *Eucalyptus* species. Interestingly, the expression levels of these proteins in the callus tissues (A2 and B2) seemed to have no difference in the two *Eucalyptus* species. Further, 27 and 4 DEPs shared by the two *Eucalyptus* species were analyzed to be enriched in the pathways of “photosynthesis” (ko00195) and “two-component system” (ko02020), respectively.

### Somatic embryogenesis potential associated proteins

We next compared the DEPs with different expression patterns in the early SE process of the two *Eucalyptus* species and identified 883 DEPs (599 upregulated and 284 downregulated) specifically deregulated in *E. camaldulensis* (Fig. 3A, Table S5), including 12 TFs, 20 RPs, 4 histone, 7 ZFPs, 7 ABC transporter proteins, 6 glutathione transferases, and 10 glucosyltransferases (Table 1). The upregulated TFs only in the SE of *E. camaldulensis* were XP_010055248.1 (BZIP16), XP_010060108.1 (GATA18), XP_018731692.1 (WRKY40), XP_010031175.1 (BIM1), XP_010048982.1 (HY5), XP_018730947.1 (SOX30), XP_010055972.1 (TGA2.3), XP_010036354.1 (TGA2.3) and XP_010052424.1 (VOZ1), while the downregulated TFs specifically identified in the SE of *E. camaldulensis* included ERF113, GT2 and XP_010032813.1 (transcription factor Pur-alpha 1). In addition, 3 cell division cycle proteins (CDCs) and 4 WD repeat-containing proteins were specifically upregulated in the SE of *E. camaldulensis* (Table 2, Table S5). It is notable that 15 DEPs were found with opposite expression patterns in the SE of the two *Eucalyptus* species (Table 2), such as XP_010043384.1 (stress-response A/B barrel domain-containing protein HS1), XP_010063266.1 (fasciclin-like arabinogalactan protein 1), XP_010060812.1 (probable glutathione S-transferase) and XP_010057953.1 (FT-interacting protein 1). Among these 15 DEPs, 7 were deregulated in the callus tissues of the two *Eucalyptus* species, indicating that they might be responsible for the high SE potential of *E. camaldulensis* (Table 2). In addition, another 73 proteins were found with differential expression (37 upregulated and 36 downregulated proteins) in A2 and B2 (Table 2), such as XP_010024431.1 (endo-1,3;1,4-beta-D-glucanase), XP_010067225.1 (ABC transporter G family member 6) and XP_018731692.1 (WRKY transcription factor 40). These proteins might be related to the high capacity of SE for *Eucalyptus*, however, their functions and the molecular regulatory mechanisms require further experiments to be explored.

**Table 2:** Proteins associated with high somatic embryogenesis potential of *Eucalyptus*

PriteinID	*E. camaldulensis*			*E. grandis* x *urophylla*			A2 vs B2^a^			Annotation
	log2FC	adj_pvalue	Regulation	log2FC	adj_pvalue	Regulation	log2FC	adj_pvalue	Regulation	
XP_010033729.1	1.53	4.73E-08	Up	-1.09	1.10E-05	Down	1.17	3.60E-05	Up	linoleate 13S-lipoxygenase 3–1, chloroplastic
XP_010057953.1	1.47	3.38E-02	Up	-1.76	1.52E-02	Down	2.24	1.24E-02	Up	FT-interacting protein 1
XP_010028309.1	1.28	2.02E-05	Up	-1.84	1.59E-07	Down	1.22	3.16E-04	Up	salicylic acid-binding protein 2
XP_010060812.1	1.69	1.58E-07	Up	-1.14	5.81E-05	Down	1.80	1.12E-06	Up	probable glutathione S-transferase
XP_010059887.1	2.95	1.51E-04	Up	-3.59	7.22E-05	Down	3.48	5.96E-04	Up	isoflavone 3'-hydroxylase
XP_010031113.1	2.43	8.95E-09	Up	-1.63	6.57E-06	Down	0.25	5.83E-01	NC	peroxidase 4-like
XP_010024060.1	3.62	1.90E-13	Up	-1.03	2.15E-04	Down	0.68	2.78E-02	NC	uncharacterized protein LOC104414606
XP_010045407.1	2.49	9.97E-07	Up	-1.86	7.48E-05	Down	0.71	1.79E-01	NC	peroxidase 4-like
XP_010046552.1	1.19	2.45E-03	Up	-1.49	3.25E-04	Down	0.75	2.60E-02	NC	2-alkenal reductase (NADP( +)-dependent)
XP_010056500.1	1.02	9.78E-03	Up	0.51	2.01E-01	NC	-2.08	7.20E-05	Down	3-hydroxyisobutyryl-CoA hydrolase 1 isoform X3
XP_018727807.1	1.55	2.70E-02	Up	-0.04	9.64E-01	NC	-1.97	2.32E-02	Down	thaumatin-like protein 1
XP_010043457.1	1.29	6.78E-04	Up	0.72	2.63E-02	NC	-1.97	1.37E-05	Down	uncharacterized protein LOC104432666
XP_010047147.1	1.18	2.38E-02	Up	0.39	4.75E-01	NC	-1.93	3.16E-03	Down	chitotriosidase-1
XP_010069582.1	1.68	2.56E-04	Up	0.85	4.84E-02	NC	-1.90	4.82E-04	Down	adrenodoxin-like protein, mitochondrial
XP_010062408.1	1.26	1.44E-02	Up	0.93	7.23E-02	NC	-1.40	2.66E-02	Down	protein AIG1
XP_010025044.1	1.87	2.30E-09	Up	0.18	4.09E-01	NC	-1.10	1.03E-04	Down	probable aldo–keto reductase 1
XP_010050124.1	1.24	1.73E-03	Up	0.69	7.24E-02	NC	-1.04	2.61E-02	Down	protein S-acyltransferase 24
XP_010055498.1	2.01	2.71E-05	Up	1.14	5.35E-02	NC	1.01	4.93E-02	Up	uncharacterized protein LOC104443686 isoform X1
XP_018715473.1	1.73	9.53E-05	Up	0.95	1.87E-02	NC	1.05	3.05E-02	Up	uncharacterized protein LOC104416785
XP_018731692.1	1.23	5.18E-04	Up	-0.86	9.72E-03	NC	1.05	9.14E-03	Up	probable WRKY transcription factor 40
XP_010024431.1	2.31	0.00E + 00	Up	0.42	3.17E-03	NC	1.07	3.15E-07	Up	endo-1,3;1,4-beta-D-glucanase
XP_010029034.1	1.38	2.36E-05	Up	0.25	3.95E-01	NC	1.07	2.73E-03	Up	lysine histidine transporter 1
XP_010060835.1	1.91	1.08E-07	Up	0.93	1.48E-03	NC	1.10	1.40E-03	Up	beta-amyrin 28-oxidase
XP_010051591.1	1.48	6.68E-05	Up	0.22	5.40E-01	NC	1.11	6.72E-03	Up	isopentenyl-diphosphate Delta-isomerase I
XP_010027995.1	1.93	4.55E-05	Up	0.62	1.40E-01	NC	1.13	2.71E-02	Up	LIM domain-containing protein WLIM1
XP_018726486.1	1.27	2.53E-04	Up	0.11	7.47E-01	NC	1.17	3.30E-03	Up	calcium-transporting ATPase 8, plasma membrane-type isoform X2
XP_010052498.1	1.55	5.52E-06	Up	0.65	2.63E-02	NC	1.22	8.46E-04	Up	ATP-dependent 6-phosphofructokinase 2
XP_010060536.1	1.77	1.72E-05	Up	0.20	6.03E-01	NC	1.31	3.35E-03	Up	stem-specific protein TSJT1
XP_010067225.1	1.42	1.26E-04	Up	-0.80	1.96E-02	NC	1.32	1.84E-03	Up	ABC transporter G family member 6
XP_010070437.1	6.06	0.00E + 00	Up	0.67	6.26E-02	NC	1.34	2.60E-03	Up	2-alkenal reductase (NADP( +)-dependent)
XP_010066562.1	1.88	1.45E-10	Up	-0.78	1.61E-04	NC	1.34	1.09E-06	Up	probable S-adenosylmethionine-dependent methyltransferase At5g38780
XP_010026302.1	2.06	3.73E-05	Up	0.74	1.13E-01	NC	1.37	9.19E-03	Up	uncharacterized protein LOC104416642 isoform X1
XP_010052253.1	1.89	1.30E-03	Up	0.56	2.63E-01	NC	1.41	2.14E-02	Up	OPA3-like protein
XP_010036163.1	1.32	6.93E-03	Up	0.56	2.59E-01	NC	1.42	1.71E-02	Up	mitochondrial import receptor subunit TOM9-2
XP_010036356.1	1.83	4.12E-04	Up	0.07	8.88E-01	NC	1.43	2.16E-02	Up	pentatricopeptide repeat-containing protein At1g80270, mitochondrial
XP_010062288.1	2.39	3.98E-11	Up	0.93	1.48E-04	NC	1.51	2.08E-06	Up	centromere protein V
XP_010034975.1	2.15	5.19E-05	Up	0.48	2.59E-01	NC	1.51	3.97E-03	Up	uncharacterized protein LOC104424307
XP_010036195.1	1.37	6.07E-03	Up	-0.58	2.44E-01	NC	1.56	9.94E-03	Up	short-chain type dehydrogenase/reductase
XP_010066560.1	2.46	5.25E-07	Up	-0.04	9.21E-01	NC	1.57	1.35E-03	Up	probable S-adenosylmethionine-dependent methyltransferase At5g37990
XP_010061568.1	1.48	4.01E-04	Up	0.28	4.87E-01	NC	1.59	1.23E-03	Up	protein odr-4 homolog
XP_010044325.1	2.50	1.44E-04	Up	0.38	5.40E-01	NC	1.62	2.57E-02	Up	uncharacterized protein LOC104433329
XP_010047608.1	1.30	1.90E-03	Up	0.00	9.99E-01	NC	1.71	9.55E-04	Up	uncharacterized protein LOC104436511
XP_010041421.1	4.02	2.79E-10	Up	-0.07	7.84E-01	NC	1.72	5.31E-06	Up	uncharacterized protein LOC104430386
XP_010066967.1	2.91	1.92E-04	Up	0.92	1.55E-01	NC	1.75	3.29E-02	Up	zinc finger CCCH domain-containing protein 17
XP_010065959.1	1.48	1.14E-02	Up	0.35	5.74E-01	NC	1.79	1.25E-02	Up	uncharacterized protein LOC104453135
XP_010060762.1	1.18	4.81E-02	Up	0.16	8.31E-01	NC	1.81	1.58E-02	Up	zinc finger BED domain-containing protein RICESLEEPER 1-like
XP_010057715.1	2.00	8.42E-05	Up	-0.64	1.58E-01	NC	1.85	1.32E-03	Up	geraniol 8-hydroxylase
XP_010024749.1	2.17	2.74E-13	Up	-0.26	3.91E-02	NC	1.86	2.16E-10	Up	major allergen Pru ar 1
XP_010034146.1	1.65	2.00E-03	Up	-0.07	9.12E-01	NC	1.91	3.33E-03	Up	pentatricopeptide repeat-containing protein At3g56030-like
XP_010070074.1	1.35	1.35E-03	Up	-0.36	5.44E-01	NC	1.97	2.14E-04	Up	glucose-6-phosphate 1-dehydrogenase 4, chloroplastic
XP_010031250.1	1.73	6.56E-04	Up	-0.09	8.68E-01	NC	2.04	8.27E-04	Up	UDP-glycosyltransferase 89B1
XP_010069584.1	1.23	1.00E-02	Up	-0.31	5.42E-01	NC	2.18	3.50E-04	Up	uncharacterized protein LOC104456478
XP_010024433.1	2.13	3.11E-10	Up	0.49	2.61E-02	NC	2.23	5.69E-09	Up	endo-1,3;1,4-beta-D-glucanase
XP_010049304.1	3.30	6.42E-04	Up	0.72	4.41E-01	NC	2.42	2.92E-02	Up	pectinesterase/pectinesterase inhibitor PPE8B
XP_018731179.1	-1.11	2.74E-02	Down	1.34	1.08E-02	Up	-0.73	2.77E-01	NC	glycerophosphodiester phosphodiesterase GDPDL3
XP_018727022.1	-1.38	1.37E-03	Down	1.02	1.64E-02	Up	0.42	4.79E-01	NC	12-oxophytodienoate reductase 3
XP_010063266.1	-1.75	2.37E-03	Down	1.41	1.36E-02	Up	-1.04	1.38E-01	NC	fasciclin-like arabinogalactan protein 1
XP_010026892.1	-2.03	4.68E-03	Down	1.92	7.87E-03	Up	-0.88	3.61E-01	NC	probable pectate lyase 18
XP_010050122.1	-1.43	7.89E-03	Down	1.25	2.25E-03	Up	-2.82	2.46E-04	Down	heparanase-like protein 3 isoform X1
XP_010043384.1	-1.85	1.20E-02	Down	1.12	3.30E-02	Up	-1.83	4.26E-02	Down	stress-response A/B barrel domain-containing protein HS1
XP_010054106.1	-19.37	1.34E-02	Down	3.67	6.62E-01	NC	-19.61	4.15E-02	Down	secretory carrier-associated membrane protein 4 isoform X1
XP_010029197.1	-4.07	1.83E-05	Down	-0.49	5.50E-01	NC	-3.64	5.26E-04	Down	2-methylene-furan-3-one reductase
XP_010035550.1	-1.07	2.58E-05	Down	-0.19	3.31E-01	NC	-2.70	3.49E-09	Down	stem-specific protein TSJT1 isoform X2
XP_010045004.1	-3.41	4.36E-09	Down	0.65	1.11E-01	NC	-2.61	4.85E-06	Down	DNA-damage-repair/toleration protein DRT100
XP_010032296.1	-3.26	2.27E-06	Down	-0.87	8.52E-02	NC	-2.61	3.37E-04	Down	NADPH:quiNC oxidoreductase
XP_010029700.1	-1.90	1.47E-04	Down	0.83	1.29E-01	NC	-2.45	1.40E-03	Down	histone H1
XP_010029991.1	-2.37	1.94E-09	Down	-0.35	1.65E-01	NC	-2.00	5.59E-07	Down	gamma-glutamyl peptidase 5
XP_010037786.1	-1.45	9.29E-03	Down	0.06	9.23E-01	NC	-1.94	5.17E-03	Down	putative receptor protein kinase ZmPK1
XP_010067858.1	-1.71	5.96E-05	Down	0.65	8.14E-02	NC	-1.90	1.54E-04	Down	uncharacterized protein LOC104454644
XP_010053269.1	-1.30	2.57E-04	Down	0.27	4.28E-01	NC	-1.86	3.39E-05	Down	probable glutathione peroxidase 8
XP_010036808.1	-2.75	6.99E-08	Down	-0.66	8.68E-02	NC	-1.80	2.86E-04	Down	CASP-like protein 1D1
XP_018733288.1	-1.43	4.53E-06	Down	-0.05	8.86E-01	NC	-1.79	1.13E-04	Down	uncharacterized protein LOC104454184
XP_010035642.1	-1.09	1.04E-07	Down	-0.36	2.26E-02	NC	-1.76	1.02E-09	Down	universal stress protein PHOS34-like isoform X1
XP_010036760.1	-1.11	1.43E-02	Down	-0.07	8.89E-01	NC	-1.74	2.09E-03	Down	cysteine-rich repeat secretory protein 55
XP_010049875.1	-1.12	2.08E-02	Down	0.24	6.34E-01	NC	-1.73	4.98E-03	Down	protein ENHANCED DISEASE RESISTANCE 2-like isoform X2
XP_010060626.1	-1.27	1.25E-05	Down	0.21	4.27E-01	NC	-1.69	2.86E-06	Down	D-amino-acid transaminase, chloroplastic
XP_010026751.1	-1.38	7.59E-07	Down	0.08	7.54E-01	NC	-1.61	1.14E-06	Down	isoflavone reductase-like protein isoform X1
XP_010041384.1	-2.07	9.97E-06	Down	-0.77	5.03E-02	NC	-1.59	1.40E-03	Down	protein SRC2 homolog
XP_010053918.1	-1.69	7.45E-03	Down	0.43	3.40E-01	NC	-1.53	5.09E-03	Down	flavin-containing monooxygenase FMO GS-OX5
XP_010044557.2	-1.19	4.95E-03	Down	0.25	5.68E-01	NC	-1.53	3.00E-03	Down	uncharacterized protein LOC104433492
XP_010053674.1	-1.38	2.12E-05	Down	0.43	1.37E-01	NC	-1.47	9.56E-05	Down	( +)-neomenthol dehydrogenase
XP_010060965.1	-1.05	3.27E-03	Down	-0.15	6.53E-01	NC	-1.44	1.03E-03	Down	isoflavone reductase-like protein
XP_010066016.1	-1.39	3.59E-05	Down	-0.67	2.80E-02	NC	-1.42	2.50E-04	Down	methylcrotonoyl-CoA carboxylase beta chain, mitochondrial
XP_010062143.1	-2.56	1.12E-08	Down	-0.79	9.21E-03	NC	-1.38	9.37E-04	Down	uncharacterized protein LOC104449618 isoform X1
XP_010046044.1	-2.21	4.18E-06	Down	-0.08	8.58E-01	NC	-1.35	6.26E-03	Down	plasma membrane-associated cation-binding protein 2
XP_010051889.1	-1.21	8.38E-06	Down	-0.05	8.98E-01	NC	-1.27	1.63E-03	Down	uncharacterized protein LOC104440663
XP_010053265.2	-1.44	1.78E-04	Down	0.03	9.44E-01	NC	-1.21	5.14E-03	Down	probable xyloglucan endotransglucosylase/hydrolase protein 16
XP_010053670.1	-1.35	3.28E-04	Down	0.26	3.80E-01	NC	-1.19	7.65E-03	Down	( +)-neomenthol dehydrogenase
XP_010061259.1	-1.17	3.47E-04	Down	-0.58	5.76E-02	NC	-1.14	2.73E-03	Down	low-temperature-induced cysteine proteinase
XP_010054042.1	-1.09	1.79E-03	Down	-0.77	2.53E-02	NC	-1.09	8.64E-03	Down	60S ribosomal protein L13-2
XP_010045283.1	-1.80	4.59E-09	Down	-0.03	9.14E-01	NC	-1.08	1.26E-04	Down	serine carboxypeptidase-like 51
XP_010047621.1	-1.31	1.99E-03	Down	-0.64	1.24E-01	NC	-1.03	4.05E-02	Down	60S ribosomal protein L31
XP_010036132.1	-1.39	3.34E-11	Down	-0.71	4.11E-06	NC	-1.03	2.08E-07	Down	alcohol dehydrogenase class-3
XP_010026802.1	-1.11	1.40E-03	Down	-0.85	1.26E-02	NC	-1.01	1.36E-02	Down	60S ribosomal protein L7a-1
XP_010051871.1	-1.36	8.88E-05	Down	-0.97	2.16E-03	NC	1.14	2.77E-03	Up	reticuline oxidase-like protein
XP_010056883.1	-1.68	2.45E-03	Down	-0.92	1.30E-01	NC	2.10	4.74E-03	Up	uncharacterized protein At2g34460, chloroplastic isoform X1

^a^A2: callus tissue of *E. camaldulensis*

B2: callus tissue of *E. grandis* x *urophylla*

### miRNA-mRNA-protein regulation networks in the early somatic embryogenesis of *Eucalyptus*

We next investigated the miRNA-mRNA-protein regulation networks in the early SE of *Eucalyptus* using the miRNA sequencing, RNA-seq and proteomics data. There were 330 upregulated and 218 downregulated genes/proteins identified by both RNA-seq and proteomics in the early SE of *E. camaldulensis* (Fig. 3C). Next, eight miRNAs were found with opposite expression patterns of the deregulated genes/proteins in *E. camaldulensis* (Table 3). Among them, egd-N-miR278-5p and egd-N-miR45-3p were predicted to target the proteins XP_010041937.1 (sodium/hydrogen exchanger 2) and XP_010067725.1 (aspartyl protease AED3), respectively. The downregulation of egd-miR164 might be the reason of the upregulation of Eucgr.J00040 and XP_010043574.1 (UDP-glucuronic acid decarboxylase 6) (Table 3). In *E. grandis* x *urophylla*, there were 324 upregulated and 219 downregulated genes/proteins identified by both RNA-seq and proteomics in the early SE of *E. camaldulensis* (Fig. 3D). Next, we found 11 miRNAs (e.g., egd-miR169, egd-miR396, egd-miR535) can regulate 10 deregulated genes/proteins in *E. grandis* x *urophylla* (Table 3), including XP_018717960.1 (UDP-glucosyl transferase 73B2-like) and XP_010070615.1 (probable indole-3-acetic acid-amido synthetase GH3.1). Among these miRNAs, we found egd-N-miR230-5p might regulate the expression of XP_010055291.1 (E3 ubiquitin-protein ligase RNF14) in the early SE of *E. grandis* x *urophylla* (Table 3). The results generated by our omics studies strongly supported the regulatory networks of miRNAs during the SE of *Eucalyptus*. Notably, the miRNAs and their target genes varied between *E. camaldulensis* and *E. grandis* x *urophylla*, indicating their association with the somatic embryogenesis potential in *Eucalyptus*, but future experiments are needed.

**Table 3:** Proteins regulated by the miRNAs during the SE of *Eucalyptus*

Eucalytpus	miRNA	log2FC	FDR	Protein	log2FC	adj_pvalue	Gene	log2FC	FDR	Protein description
*E. camaldulensis*	egd-N-miR278-5p	2.65	2.31E-08	XP_010041937.1	-2.68	4.87E-04	Eucgr.B01758	-2.09	3.52E-07	sodium/hydrogen exchanger 2
	egd-N-miR45-3p	1.89	4.40E-06	XP_010067725.1	-2.09	2.41E-07	Eucgr.G03227	-2.23	6.08E-08	aspartyl protease AED3
	egd-N-miR1-3p	-1.79	6.95E-06	XP_010045128.1	2.09	1.20E-09	Eucgr.B03780	5.08	6.94E-28	protein argonaute 4
	egd-N-miR86-3p	-3.93	9.42E-19	XP_010050073.1	4.35	1.59E-09	Eucgr.A01561	3.56	1.35E-16	AT-hook motif nuclear-localized protein 7
	egd-miR160c-5p	-12.49	5.84E-49	XP_010060919.1	1.39	2.88E-09	Eucgr.A02778	1.45	3.96E-04	tripeptidyl-peptidase 2 isoform X1
	egd-miR164	-2.98	5.44E-13	XP_010043574.1	1.98	7.34E-03	Eucgr.J00040	1.46	3.73E-04	UDP-glucuronic acid decarboxylase 6
	egd-miR395	-10.61	6.61E-34	XP_010024218.1	2.35	1.91E-06	Eucgr.H03408	1.92	2.79E-06	ATP sulfurylase 1, chloroplastic
	egd-miR530-5p	-5.10	6.60E-26	XP_010047053.1	2.98	1.74E-08	Eucgr.C00240	2.00	9.97E-07	uncharacterized protein LOC104436016
*E. grandis* x *urophylla*	egd-N-miR139-5p	1.12	7.70E-03	XP_018721074.1	-1.02	2.74E-06	Eucgr.K00558	-1.19	5.40E-03	protoporphyrinogen oxidase 1, chloroplastic
	egd-N-miR174-5p	2.57	2.21E-07	XP_010062805.1	-2.69	2.87E-02	Eucgr.F03259	-2.48	2.45E-09	putative serine/threonine-protein kinase
	egd-N-miR230-5p	1.59	3.55E-04	XP_010055291.1	-1.47	2.81E-02	Eucgr.E00258	-1.88	5.45E-06	E3 ubiquitin-protein ligase RNF14
	egd-N-miR30-5p	1.29	1.26E-03	XP_010036653.1	-1.28	8.72E-05	Eucgr.K02010	-1.35	1.24E-03	putative calcium-transporting ATPase 11, plasma membrane-type isoform X1
	egd-N-miR300-5p	3.51	1.28E-10	XP_010044969.1	-1.70	1.04E-03	Eucgr.B03634	-3.32	1.39E-14	metal tolerance protein 4
	egd-miR169af-5p	-6.02	2.99E-24	XP_010032744.1	1.06	2.76E-03	Eucgr.J01628	1.17	6.44E-03	cytochrome c oxidase assembly protein COX11, mitochondrial isoform X1
	egd-miR169r-5p	-5.01	1.50E-25	XP_018717960.1	2.78	1.65E-06	Eucgr.I01125	2.63	2.78E-10	UDP-glucosyl transferase 73B2-like
	egd-miR169r-5p	-5.01	1.50E-25	XP_010031608.1	1.57	5.06E-04	Eucgr.J00605	1.17	4.98E-03	uncharacterized protein LOC104421386
	egd-miR396a-5p	-3.24	9.05E-15	XP_010070615.1	5.08	1.43E-08	Eucgr.H02264	10.08	5.93E-66	probable indole-3-acetic acid-amido synthetase GH3.1
	egd-miR535a-5p	-1.74	1.26E-05	XP_018730129.1	1.56	2.49E-04	Eucgr.E01067	2.27	9.01E-08	glucan endo-1,3-beta-glucosidase 11
	egd-miR535b-5p	-1.75	1.18E-05							

### qRT-PCR validation

**Fig. 4:**
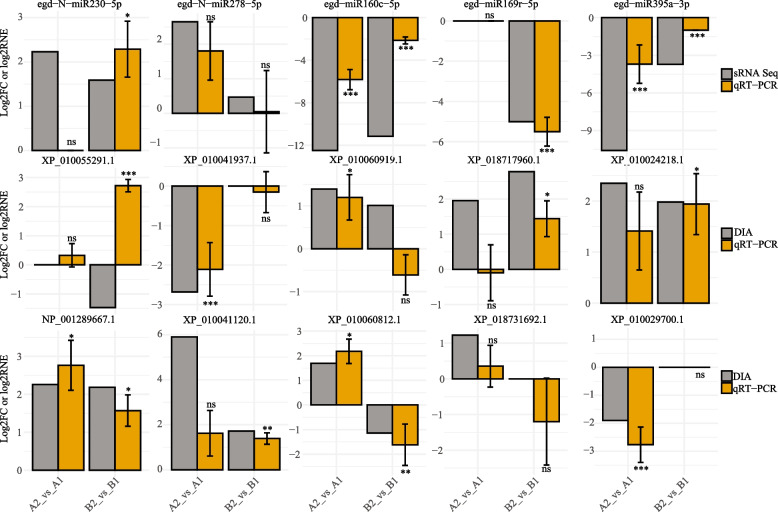
qRT-PCR validation. The genes from the horizontal panel in the middle are targets of the miRNAs above. Triplicate reactions were performed for each gene/miRNA in each sample. Log2FC and log2RNE were used to show the expression changes of miRNAs/proteins in the comparisons. The error bars were present for the mean ± SD of log2RNE of candidate miRNAs/proteins in the comparisons. A1: stem tissue of *E. camaldulensis*; A2: callus tissue of *E. camaldulensis*; B1: stem tissue of *E. grandis* x *urophylla*; B2: callus tissue of *E. grandis* x *urophylla*. ns: not significant; *: *p* < 0.05; **: *p* < 0.01; ***: *p* < 0.001

We used qRT-PCR to validate the expression changes of miRNA and protein candidates involved in the early SE and related to the SE potential of *Eucalyptus*. We randomly selected 5 miRNAs and 10 protein encoding genes (including the target genes of the selected miRNAs) for the qRT-PCR experiment and the primers can be seen in Table S6. The qRT-PCR experiment first confirmed the expression changes of 10 proteins during the SE of Eucalyptus and 14 out of 20 events were agreed by both DIA and qRT-PCR (Fig. 4), such as NP_001289667.1, XP_010024218.1 and XP_010060812.1. Then, the qRT-PCR experiment provided evidence for the regulation of miRNA ~ protein networks, such as egd − N − miR278 − 5p ~ XP_010041937.1, egd-miR160c-5p ~ XP_010060919.1 and egd-miR395 ~ XP_010024218.1 in *E. camaldulensis*, and egd − miR169r − 5p ~ XP_018717960.1 and miR395 ~ XP_010024218.1 in *E. grandis* x *urophylla* (Fig. 4). Overall, 20 out of 30 (66.7%) events were agreed by the small RNA sequencing/DIA proteomics and qRT-PCR. High agreement of protein expression patterns and miRNA ~ protein regulation pairs by sequencing and qRT-PCR indicate that they might be associated with the early SE and SE potential of Eucalyptus. However, their functions and regulation require further experiments to be explored.

## Discussion

In this study we analysed the proteome profiles of stem and tissue-culture induced callus tissues of two *Eucalyptus* species and aimed to identify proteins involved in early SE and related to the SE potential of *Eucalyptus*. Some known SE related proteins were found in this study, such as ARF19, WOX8, PIN1 and aquaporins (Fig. 3B). Among them, ARF19, together with ARF7, has been reported to play a key role in the dedifferentiation process and to regulate the expression of lateral organ boundaries domain 29 (LBD29) which controls both in vivo and in vitro dedifferentiation of *Arabidopsis* cells [36, 37]. In addition, ARF19 can biologically function in responding to auxin, whose effects in SE have been presented extensively in many studies [38–40], and brassinosteroid [9], which are a class of plant steroid hormones and can promote the cell elongation, cell division, and dedifferentiation throughout the plant cell life cycle [41, 42]. In this study, we found two ARF19 proteins (XP_010048533.1 and XP_010049373.1) commonly upregulated in the SE of *E. camaldulensis* and *E. grandis* x *urophylla* (Fig. 3B), however, LBD29 has not been annotated for *Eucalyptus* genome and was not found in this study. WOX8 (NP_001289667.1) is another important upregulated protein found in the SE of both *E. camaldulensis* and *E. grandis* x *urophylla* (Fig. 3B). WOX gene family is a large class of homeodomain TFs that are involved in the early phase of embryogenesis and lateral organ development in plants [43]. The WOX members WOX2, WOX8 and WOX9 are important cell fate regulators of early pre-embryos [43, 44]. It seems that WOX genes are induced at the first stages of SE and the expression of WUS/WOX genes can increase the efficiency of SE induction and lead to the formation of somatic embryos without adding hormones [45, 46]. For example, the overexpression of *MtWOX9-1* improves the SE efficiency and is linked with the increase of AGL15 and AGL8 [46]. In this study PIN1 gene was annotated to encode the auxin efflux carrier component 1 and can be triggered by the synthetic auxin 2,4-D in immature embryos of maize and *Arabidopsis* at early induction [47]. Our results confirmed the upregulation of WOX8 and PIN1 in the early SE of both *Eucalyptus* species. Interestingly, we observed the downregulation of aquaporins (Fig. 3B) in the SE of *Eucalyptus*, but evidence for aquaporin in SE is very limited. Our results indicated that these proteins might be markers of SE and that can be used in the *Eucalyptus* breeding program.

The comparison of DEPs in the early SE of the two *Eucalyptus* species revealed some proteins related to the SE potential (Table S5), such as XP_010024200.1 (SERK2), XP_018731692.1 (WRKY40), ZFPs and ABC transporters. SERK family proteins and genes have been reported to participate in the oxidative and pathogen stress signalling, embryogenic competence and development [7]. These have been experimentally confirmed in many other plant species, such as soybean [48], rice [49], alfalfa [50], maize [51], pineapple [52] and cotton [53]. Interestingly, under the 2,4-D induction *VvSERK1* and *VvSERK2* were documented to be decreased during the secondary embryogenesis in grapevine [54]. However, in this study we found the upregulation of SERK2 only in the SE of *E. camaldulensis* (Table S5), and the upregulation of SERK2 under 2,4-D treatment during SE was also reported in *Arabidopsis* [55] and pineapple [52]. This can be explained by that SERK could only be detected transiently in the zygotic embryo up to the early globular stage [56]. Considering that SERK2 was only upregulated in the early stage of SE in *E. camaldulensis*, it might be related to the high SE potential for *Eucalyptus*. WRKY proteins are plant-specific transcription factors and have been widely reported to play important roles in various physiological processes and metabolisms, particularly in biotic and abiotic stresses [57]. The expression of WRKY genes are inducible during the SE of many plants, such as papaya [58], cotton [53], *Panax ginseng* [59] and *Arabidopsis* [60], indicating its crucial role in the SE process. More importantly, in our transcriptome study the WRKY TF genes were specifically upregulated in the early SE of *E. camaldulensis* [2]. Like WRKY TF, ABC transporter proteins and genes were specifically upregulated in the callus compared to the stem of *E. camaldulensis* (Table 1) [2]. Some members of the ABC transporter family have been confirmed to mediate the auxin transport, like ABCB1, ABCB4, ABCB14, ABCB19, and ABCB21 [61, 62]. ABCB21 and ABCB28 were found with high expression in the callus of *E. camaldulensis* (Table S5). It is interesting that three zinc finger CCCH domain-containing proteins were upregulated in the early SE process of the two *Eucalyptus* species, and more ZFPs were specifically upregulated in the SE of *E. camaldulensis* (Table S5), including three CCCH domain-containing and three BED domain-containing ZFPs. The CCCH ZFP was reported to be induced at SE in suspension culture and enhanced by the treatment by about 50 times in cucumber [63]. The functions of the ZFPs and other *E. camaldulensis* specifically deregulated proteins in SE require further experiments to be explored.

Investigation of miRNA-mRNA-protein network can improve our understanding of the molecular regulation during the early SE of *Eucalyptus*. Using the same material, we identified 179 miRNAs (e.g., miR156, miR159, miR160, miR164, miR166, miR169, miR171, miR399, and miR482) commonly deregulated in the two species and 148 miRNAs (e.g., miR159c-3p, miR167a-5p, miR397a-3p, miR397c-5p, miR397d-3p, miR397d-5p, N-miR1-5p, N-miR5-5p, miR482b-3p, N-miR3-3p, miR156a-3p, N-miR40-3p, N-miR18-5p) miRNAs specific to *E. camaldulensis* [28]. In this study we analysed the regulatory targets of some miRNAs on both mRNA and protein levels in the early SE of *Eucalyptus* (Table 3), such as miR160 ~ TPP2, miR164 ~ UXS2, miR169 ~ COX11 and miR535 ~ Eucgr.E01067. Due to the missing of some genes for the proteins, some of the miRNA targets identified in this study might be different from our small RNA and transcriptome sequencing studies [2, 28]. For example, ARF18 (Eucgr.K01240) is a target of miR160 in our RNA studies, but its protein is not found in the Eucalyptus genome. However, based on the DIA results we found TTP2 is a target of miR160 (Table 3). The TPP2 gene is widely expressed in many tissue types of *Arabidopsis*, as well as other plant genomes [64]. It is a serine protease of the proteasome pathway and may function with the 20S proteasome to degrade oxidized proteins generated by environmental stress [64, 65]. UXS2 is necessary for the biosynthesis of the core tetrasaccharide and has been reported to catalyse the NAD-dependent decarboxylation of UDP-glucuronic acid to UDP-xylose [66, 67]. Like TPP2 and UXS2, the functions of COX11 and Eucgr.E01067 have not been reported in SE or dedifferentiation in plants, however, their abnormal expression and regulation relationships indicated that they might play key roles in this process.

## Conclusions

In conclusion, we compared the proteome profiles of stem and callus tissues of two Eucalyptus species and identified some proteins involved in the early SE of Eucalyptus, including previously reported proteins ARF19, WOX8 and PIN1. Further, we found that SERK2, ABC transporter proteins and ZFPs might be associated with the high regeneration ability of *Eucalyptus*, due to their diverse expression patterns in the early SE of two Eucalyptus species. Some miRNA ~ protein regulation networks provided new insights of the molecular mechanisms involved in the early SE of *Eucalyptus*, such as miR160 ~ TPP2, miR164 ~ UXS2, miR169 ~ COX11 and miR535 ~ Eucgr.E01067. Further, we used qRT-PCR to validate the protein expression on gene level and it showed high agreement with the DIA proteomics approach. This is the first time to investigate the protein changes during the early SE of *Eucalyptus* and the findings will improve our understanding of the proteins involved in this process. The miRNA regulatory networks and high regeneration ability associated proteins might be our future research focuses.

## Supplementary Information

Below is the link to the electronic supplementary material.

**Additional file 1** **Table S1.** Differentially expressed proteins identified in the early SE of *E. camaldulensis*. **Table S2.** Gene Ontology enrichment analysis of DEPs in *E. camaldulensis*. **Table S3.** Differentially expressed proteins identified in the early SE of E. grandis x urophylla. **Table S4.** Gene Ontology enrichment analysis of DEPs in *E. grandis x urophylla*. **Table S5.** Specifically deregulated proteins in the early SE of E. camaldulensis. **Table S6.** qRT-PCR primers used for this study.

## Data Availability

The original files of DDA and DIA MS data can be accessed from the iProx website under the accession numbers IPX0004336001 and IPX0004336002.

## Abbreviations

SE: Somatic embryogenesis
CIM: Callus induction medium
ARF19: Auxin response factor 19
PRC1: Polycomb repressive complex 1
RGP1: UDP-arabinopyranose mutase 1
HSP17: Heat shock protein 17
SERK1: Somatic embryogenesis like receptor kinase 1
LEC1: Leafy cotyledon 1
GLB1: Galactosidase beta 1
WUS: WUSCHEL protein
CLF: Curly leaf
CDKA1: Cyclin-dependent kinase A-1
PRZ1: Transcriptional regulator prz1
STM: Homeobox protein SHOOT MERISTEMLESS
DEP: Differentially expressed protein
RP: Ribosomal protein
ZFP: Zinc finger protein
HSP: Heat shock protein
TF: Transcription factor
FA: Formic acid
DDA: Data-dependent acquisition
MIT: Maximal injection time
DIA: Data-independent acquisition
FDR: False discovery rate
GO: Gene Ontology
COG: Cluster of Orthologous Group
KEGG: Kyoto Encyclopedia of Genes and Genomes
WOX8: WUSCHEL-related homeobox 8
CDC: Cell division cycle protein
LBD29: Lateral organ boundaries domain 29
TPP2: Tripeptidyl-peptidase 2
